# NMR spectroscopy analysis reveals differential metabolic responses in arabidopsis roots and leaves treated with a cytokinesis inhibitor

**DOI:** 10.1371/journal.pone.0241627

**Published:** 2020-11-06

**Authors:** Thomas E. Wilkop, Minmin Wang, Angelo Heringer, Jaideep Singh, Florence Zakharov, Viswanathan V. Krishnan, Georgia Drakakaki

**Affiliations:** 1 Light Microscopy Core/ Department of Physiology, University of Kentucky, Lexington, KY, United States of America; 2 Department of Plant Sciences, University of California, Davis, CA, United States of America; 3 Department of Chemistry, California State University, Fresno, CA, United States of America; 4 Department of Medical Pathology and Laboratory Medicine, University of California School of Medicine, Sacramento, CA, United States of America; University of Massachusetts Amherst, UNITED STATES

## Abstract

In plant cytokinesis, *de novo* formation of a cell plate evolving into the new cell wall partitions the cytoplasm of the dividing cell. In our earlier chemical genomics studies, we identified and characterized the small molecule endosidin-7, that specifically inhibits callose deposition at the cell plate, arresting late-stage cytokinesis in arabidopsis. Endosidin-7 has emerged as a very valuable tool for dissecting this essential plant process. To gain insights regarding its mode of action and the effects of cytokinesis inhibition on the overall plant response, we investigated the effect of endosidin-7 through a nuclear magnetic resonance spectroscopy (NMR) metabolomics approach. In this case study, metabolomics profiles of arabidopsis leaf and root tissues were analyzed at different growth stages and endosidin-7 exposure levels. The results show leaf and root-specific metabolic profile changes and the effects of endosidin-7 treatment on these metabolomes. Statistical analyses indicated that the effect of endosidin-7 treatment was more significant than the developmental impact. The endosidin-7 induced metabolic profiles suggest compensations for cytokinesis inhibition in central metabolism pathways. This study further shows that long-term treatment of endosidin-7 profoundly changes, likely via alteration of hormonal regulation, the primary metabolism of arabidopsis seedlings. Hormonal pathway-changes are likely reflecting the plant’s responses, compensating for the arrested cell division, which in turn are leading to global metabolite modulation. The presented NMR spectral data are made available through the Metabolomics Workbench, providing a reference resource for the scientific community.

## Introduction

**Fig 1:**
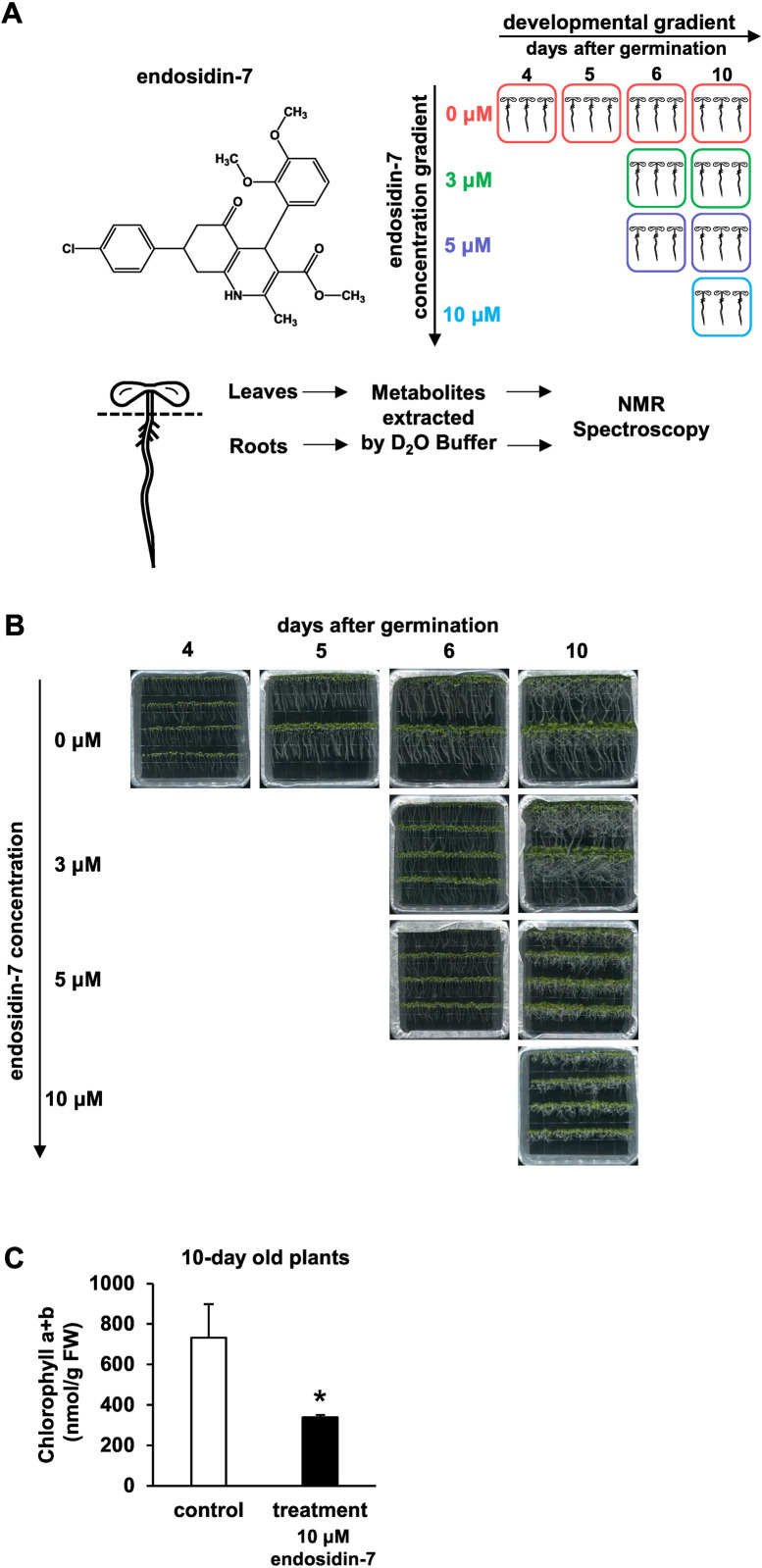
Experimental design and phenotypic responses to endosidin-7 treatments. (A) Experimental design of endosidin-7 treatment and NMR Metabolomics analysis. The molecular structure of endosidin-7 is shown on the left. Arabidopsis seedlings were treated with 0, 3, 5, and 10 μM of endosidin-7 and grown for up to 10-day old, with 4, 5, 6, 10-day old seedlings used as developmental controls. The cartoon of three seedlings represents three biological replicates; each replicate comprised pooled seedlings from one plate. Leaves and roots were extracted by deuterated water and subjected to NMR spectroscopy analysis. (B) Morphological phenotype of endosidin-7 treated arabidopsis seedlings. Images were recorded before the metabolite extraction for NMR analysis. An endosidin-7 concentration-dependent inhibition of seedling growth was shown. (C) Chlorophyll content of endosidin-7 treated leaves. Samples from endosidin-7 treated leaves (10-day old) compared with the control (10-day old). A significant reduction in the chlorophyll content of endosidin-7 treated leaves was observed. Data represent the mean ± SD (n = 4), with the asterisk indicating *p* <0.05 in the t-test.

In a large-scale chemical genetics screening of small molecules interfering with endomembrane trafficking in arabidopsis [1], a number of highly specific compound-probes were identified. Among these compounds was endosidin-7, a heterocyclic organic molecule with attributes of both flavonoid and alkaloid derivatives (**Fig 1A**), that specifically inhibits callose deposition at the division plane, which consequently leads to late-stage cytokinesis arrest [2]. Cytokinesis is a fundamental process of all life on earth and is essential for plant growth and development. The tight regulation of this process involves the coordinated accumulation of membrane material and polysaccharide deposition. It is currently hypothesized that callose integration structurally stabilizes the maturing cell plate while it transitions into a new cell wall [3–5]. Cell plate formation involves highly orchestrated vesicle accumulation, fusion, and membrane network maturation, and is supported by the temporary integration of elastic and pliable callose [6, 7]. Currently, the integration and coordination of polysaccharide deposition, in conjunction with the membrane maturation during cell plate expansion, is ill-understood [6, 7]. A detailed understanding of the plant’s metabolome in response to endosidin-7 treatment can provide insights into major metabolic fluxes during plant cytokinesis and potential compensating mechanisms to its inhibition.

The utility of endosidin-7 as a cytokinesis probe has been shown by its ability to reveal the interplay of specific vesicle populations during cell plate assembly [2, 8]. With the aid of endosidin-7, the timely pattern of vesicle contributions during cell plate expansion can be dissected to better understand their specific roles. This includes distinguishing between the early arrival of GTPase RABA2a labeled cytokinetic vesicles and the vesicle fusion mechanisms visualized by the cytokinesis-specific SNARE protein KNOLLE [2]. The removal of excess membrane material, facilitated by clathrin-coated vesicles, accompanies callose deposition. Endosidin-7 treatment reduces the amount of these vesicles, suggesting that the cell plate does not reach the excess membrane removal stage and supports the notion of a vital role for the temporal calose integration during cell plate maturation [2]. In our earlier studies in arabidopsis, we showed that endosidin-7 indirectly inhibits callose synthase activity, specifically incorporating UDP-glucose into β-1,3-glucan. Notably, the effect of endosidin-7 is specific, with no discernable differences during interphase cells in non-dividing cells. In addition, it does not affect wound-induced callose deposition or plug formation in sieve elements [2]. Consistent effects of endosidin-7 across the plant kingdom, from early diverging algae, e.g., Charophyte *Penium margaritaceum*, to higher plants, demonstrate that the pathways affected by endosidin-7 are evolutionarily conserved [9].

*Arabidopsis thaliana* L. (Heynh). is a well-established model organism employed in many studies to understand biological functions across the plant kingdom [10]. Many detailed omics studies have been carried out to map and investigate the transcriptome, proteome, and metabolome of arabidopsis during growth and development [11–13]. This combined wealth of arabidopsis knowledge makes this model plant an excellent choice for untargeted metabolite analysis. The significance of arabidopsis as a model system has led to several nuclear magnetic resonance (NMR)-based metabolomics studies with both solution and solid-state samples [14–17]. Recently developed approaches of ^1^H high-resolution magic angle spinning NMR, which are circumventing the need for elaborate sample preparation and allowing utilization of solid-state NMR spectroscopy, were employed for the study of intact arabidopsis leaves [18, 19]. NMR-based methods for studying plant metabolomics, including sample preparation protocols and data analysis approaches, are being refined continuously [20, 21], contributing to a proliferation in their utilization.

In order to understand the effect of endosidin-7 on overall plant physiology and metabolism, we performed an NMR-based metabolomics analysis. Given the metabolic differences between aerial tissues and roots, including photosynthesis, carbon assimilation, and nutrient acquisition [22–24], we investigated roots and leaves separately. Metabolites were monitored in roots and leaves of treated and non-treated arabidopsis seedlings over periods of 4–10 days. To investigate the factors affecting metabolite levels in roots and leaves upon endosidin-7 treatment, we utilized a partial least squares discriminant analysis (PLS-DA) for the classification of metabolites across developmental stages and endosidin-7 treatment concentrations. A comprehensive multivariate statistical analysis was employed to identify and quantify the metabolites differentially altered due to endosidin-7 treatment. We found that the concentrations of over 50 metabolites were affected in arabidopsis roots and leaves as a result of endosidin-7 induced cytokinesis inhibition. Additionally, our work provides an NMR-based metabolomics protocol to study the effect of small molecules on plant metabolism.

## Materials and methods

### Plant materials and metabolite extraction

Arabidopsis seedlings (Columbia) were germinated on agar media with half-strength MS basal salts and 1% sucrose. Seedlings were grown at 22°C with a 16h light cycle at ~80 μmol m^-2^ s^-1^ light intensity. Endosidin-7 was dissolved in DMSO and supplemented into the medium at concentrations of 0, 3, 5, and 10 μM. Seedlings were germinated in a vertical orientation, encouraging directional root growth, easier treatment assessment, and tissue collection. During sample harvesting, roots and leaves, including hypocotyls as indicated in **Fig 1A**, were separated by sectioning with razor blades. Metabolites were extracted following a standard procedure [20, 25]. All seedlings in one plate composed an individual biological replicate. Approximately 50 mg of root tissue and 300 mg of leaf tissue were harvested for each biological replicate. Tissue was homogenized with mortar and pestle in liquid nitrogen, and metabolites were extracted in methanol. Homogenized tissue was lysed using ice-cold 80/20 (v/v) methanol/water by v/w ratio of 3:1 in 1.5 mL tubes by vortexing/trituration and then incubated for 20 minutes on ice. Samples were centrifuged for 10 minutes at 10,000*g*, the clarified supernatant was dried in a speed-vacuum/lyophilizer, and the dried pellet was stored at -80°C. Subsequent sample preparation for NMR spectroscopy was performed on the dried samples, as described below.

For chlorophyll quantification in leaves, 10-day old seedlings, grown as described above, were used. The glucanase activity assay was performed using leaf crude extracts of 10-day old plants. Both chlorophyll and glucanase activity analyses are described below.

### Proton nuclear magnetic resonance spectroscopy

For the ^1^H NMR analysis, the aforementioned extracted samples were resuspended to a final volume of 600 μL in D_2_O, with 0.35 mM sodium trimethylsilyl-2,2,3,3-d_4_-propionate (TSP) added to each lyophilized, titrated extract for chemical shift calibration. All sample preparations were performed over two days, and samples were subsequently stored at 4°C. Quantitative ^1^H-NMR spectra were recorded at 800 MHz and 300 K on an Avance III spectrometer (Bruker Biospin, Wissembourg, France) using a 5-mm ATMA broadband inverse probe. One-dimensional ^1^H experiments, with a mild pre-saturation of water resonance, were performed with a 90° pulse angle. NMR spectra were collected over 512 transients, with an acquisition time of 2.5 s, and a relaxation delay of 1.0 s.

The spectra were processed and analyzed with Chenomx NMR Suite 8.1 software (Chenomx Inc., 2014). Fourier-transformed spectra were multiplied with an exponential weighting function corresponding to a line-broadening of 0.5 Hz. All the spectra were manually phase-corrected, baseline optimized, and their chemical shifts were referenced to TSP. The resulted spectra were analyzed using the PROFILER-Module of Chenomx, and the concentrations of selected metabolites were estimated in all the samples. The combined concentration data was used for the multivariate statistical analysis. The metabolite peaks of the processed spectra were analyzed and assigned to their chemical shifts using the built-in Chenomx and the Human Metabolome Database [26]. The assigned metabolites were compared and confirmed through chemical shift values of other NMR based metabolomics studies performed in arabidopsis [16, 18, 27] and through comparison with the Metabolomic Repository Bordeaux (MeRy-B) database [28]. The concentrations of the assigned metabolites were determined using the Chenomx software and the concentration of the internal standard TSP [16]. All the NMR spectral data were deposited in Metabolomics Workbench (accession ST001478) under project DOI: 10.21228/M85T2G.

### Statistical analysis of NMR spectroscopy datasets

Metabolite concentrations in leaf and root extracts from different experimental conditions were analyzed using multivariate statistical analyses based on previously established methods [29, 30]. A description for calculating the differential expression of metabolites between two groups can be found in the detailed protocol by Chong et al. [31]. Briefly, a linear model fit was determined for each analyte using the LIMMA package in R [32]. Lists of metabolites with the most evident differential levels between the groups (control vs. treatment; leaves vs. roots; growth periods and endosidin-7 concentration) were obtained. Significantly changed metabolites were selected via a two-step process. First, the initial data set consisted of all the metabolites for which a signal was detected for at least one feature (e.g., control group of 4-day old plants) for one condition. Second, the data from all the comparisons were combined into a single data set. The resulting combined data set consisted of metabolites exhibiting modulation for at least one experimental comparison tested. Differential measurements within groups of samples, i.e., control samples at a particular day and endosidin-7 treatment, were detected by an *F*-test. *P*-values for different analytes were transformed to compensate for multiple comparisons using the False Discovery Rate (FDR) adjustment (FC > 1.5 and *p*-value < 0.05) for multiple comparisons using the Benjamini-Hochberg procedure [33, 34]. Fold changes were derived from multivariate statistical analysis. This analysis allowed a comparison between multiple groups, and to provide a more meaningful value for fold change and adjusted *p*-values across multiple comparisons. The threshold for significance was a *p*-value < 0.05 for all tests with a fold change of (log2) > 1.5, unless otherwise stated in the specific analysis. All the analyses and plots were produced using a combination of Bioconductor and R [32, 35].

### Spectrophotometric assays

Glucanase activity assays were performed according to the protocol provided by Choudhury [36]. Leaves of 10-day old arabidopsis seedlings grown on agar plates were homogenized in liquid nitrogen with 50 mM sodium acetate buffer (pH5.2) containing 1 mM PMSF in a 1:1 w/v ratio using mortar and pestle. The homogenates were then filtered through Miracloth (MilliporeSigma, Burlington, MA, USA), and subsequently cleared by centrifugation at 1000*g* for 2 min at 4°C. The clear upper phase of the lysate was desalted by size exclusion chromatography, using a PD MiniTrap G-25 prepacked column (GE Healthcare, Chicago, IL, USA) with assay buffer as eluent. The protein content was measured by Bradford protein assay [37], and extracted proteins were used for the glucanase assay with background level estimation, as described below. A 100 μL assay mixture contained 50 μL desalted crude extract, 1 μL of DMSO or DMSO containing endosidin-7, 19 μL of DI water, and 30 μL laminarin (TCI America, Portland, OR, USA) to yield a final concentration of 15 g/L as substrate. The standard curve was established with 3.125 to 100 μg of glucose dissolved in the assay buffer. Assays were performed at 50°C for 45 min, and terminated with 900 μL 3,5-dinitrosalicylic acid reagent at 85°C for 10 min. Then absorbance was recorded at 510 nm on a spectrophotometer (UV-1700, Shimadzu, Kyoto, Japan). Background levels of reduced sugars in the assay were determined using boiled protein extracts as reference.

Leaf chlorophyll content was quantified according to an established protocol [38]. Briefly, chlorophyll was extracted from weighed arabidopsis leaves (20–40 mg) with 400 μL methanol/chloroform (2:1, v/v) for 1 h. Then 300 μL of water with 125 μL chloroform were added into the mixture to facilitate phase separation. After centrifugation at 10000 *g* for 5 min, the lower chloroform phase was air-dried and resuspended in methanol. Chlorophyll (a and b) content was calculated from the sample’s absorbance at 665nm, 652 nm, and 750 nm, using the extinction coefficient for suspension in methanol and the formula provided by Porra et al. [38].

## Results

### Phenotypic responses of endosidin-7 treated arabidopsis seedlings

Arabidopsis seedlings were grown for 4, 5, 6, and 10 days after germination with 0 μM endosidin-7 in the media and only DMSO as reference and control (**Fig 1A and 1B**). The effect of endosidin-7 was assessed by seedling growth inhibition under 3, 5, or 10 μM endosidin-7 treatment for up to 6 or 10 days (**Fig 1A and 1B**). The selected endosidin-7 concentration range was based on the previously established (by root growth inhibition) IC_50_ of 5 μM [2]. Compared to untreated controls, endosidin-7 treated seedlings exhibited, in a concentration-dependent manner, consistently shorter roots (**Fig 1B**), corroborating our earlier observations [2]. Notably, loss of gravitropism was observed in endosidin-7 treated 10-day old samples. The aerial part of the leaves was similarly affected, as indicated by its diminished growth. To assess the impact on the leaves, we measured the chlorophyll content of 10-day old seedlings treated with 10 μM endosidin-7. The leaf chlorophyll content showed a >50% reduction compared to the untreated control (**Fig 1C**), indicating a significant loss of photosynthetic activity. Given that endosidin-7 reduces plant growth, we allowed the plants to grow for 6 or 10 days to ensure the availability of sufficient harvestable material for metabolite analysis. Metabolites were extracted from leaves and roots, and NMR spectra were recorded.

### The effect of endosidin-7 treatment is greater than the developmental impact on the arabidopsis metabolomes

Generally, a difference in the NMR spectra representing the metabolite profiles was observed for the different organs, as shown by spectral excerpts of 10-day old seedlings (**S1 Fig**). A partial least squares discriminant analysis (PLS-DA) of all the untreated leaf and root samples (**S2A Fig**) underscores the prominent difference between leaf and root metabolites. Biological triplicates of control metabolomes for each time point were tightly correlated, demonstrating the consistency in the experiments and the robustness of the analysis (**S2B Fig**). In general, the NMR spectra of the leaves, in comparison with that of the roots, tend to have additional spectral features at the aromatic region and beyond (>7.00 ppm) (**S1 Fig**). Furthermore, considering all the untreated samples (all developmental stages), the metabolites of root samples cluster much tighter than that of leaves (**S2A Fig**), indicating a larger variation in the leaf samples. PLS-DA on the different developmental control metabolomes indicated a difference across the different developmental stages (**S2C Fig**, PC1 = 16.8% and 18.8% for leaves and roots, respectively).

A multivariate analysis was performed to quantify potential changes in the observed metabolites across the developmental gradient between 4–10 days after germination. The developmental data (leaves or roots) without endosidin-7 treatment at 10, 6, 5-day old samples was compared with reference to the data of 4-day old samples. A trend of potential differences was observed, and some of the metabolites passed the fold-change criteria (> 1.5); however, the changes were not statistically significant (*p*-value > 0.05). Potentially the dense population of seedlings, especially at 10 days after germination, as shown in **Fig 1B**, could account for an increase of stress-related metabolites compared to the 4-day old plants. However, the absence of statistical differences at these stages suggests that pressure under the growth conditions did not induce discernable differences in the analyzed NMR metabolome.

**Fig 2:**
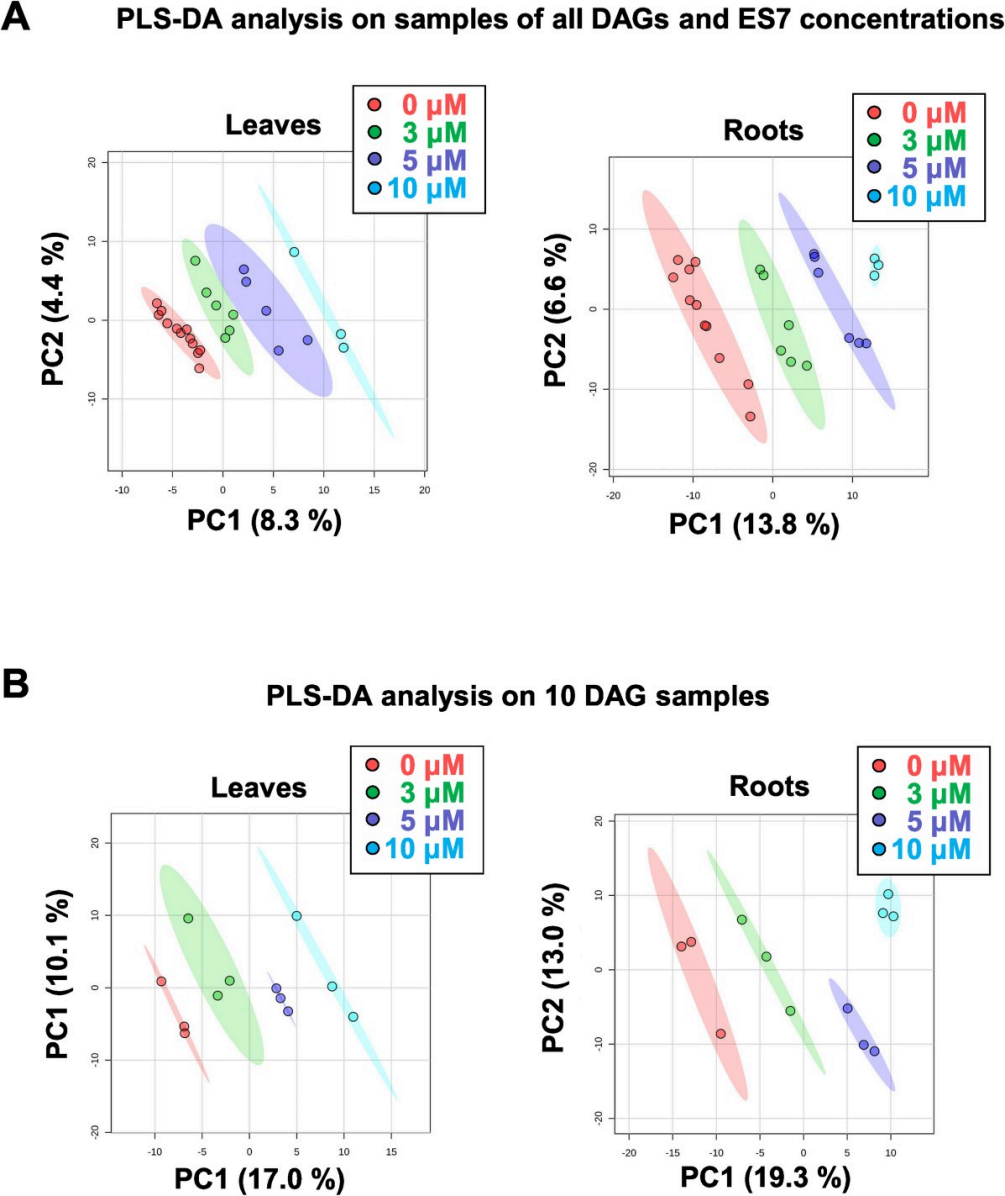
Classification of NMR based metabolomics of the endosidin-7 treated seedlings. (A) PLS-DA analysis of all NMR data. (B) PLS-DA analysis of 10-day old samples. In (A) and (B), each dot represents one biological replicate, as indicated in Fig 1A with the same color scheme. Ellipses, indicating a 95% confidence region, are shown for the classifications of biological replicates with their respective endosidin-7 concentrations.

A series of statistical analyses were applied to determine if the endosidin-7 treatment is the dominant factor contributing to the metabolite changes. In order to assess the degree of dispersion in the metabolomes across the developmental gradient and endosidin-7 treatment, we analyzed the metabolite data of all 27 samples (**Fig 1A)** for roots and leaves by PLS-DA. For both roots and leaves, PLS-DA for 0, 3, 5, and 10 μM endosidin-7 treatment were grouped tightly into areas of 95% confidence regions, marked by ellipses, and are well separated from the collective metabolome of the 12 untreated controls, across all developmental stages combined (**Fig 2A**). Even at the lowest used endosidin-7 concentration of 3 μM, no cluster overlap was observed with the control samples (**Fig 2A**, PC1 = 8.3% and 13.8% for leaves and roots, respectively). This shows that the plant growth under tissue culture settings and the developmental stage did not induce significant changes to mask the effect of endosidin-7. For the 10-day old plants metabolomes (**Fig 2B**), for 0, 3, 5, and 10 μM endosidin-7 concentrations, a clear separation of clusters was observed, accounting for the increased chemical treatment. The cross-validation of all the performed PLS-DA analysis, **S3 Fig**, with the corresponding unsupervised principal component analysis (PCA) **S4 Fig,** is detailed in the supporting information.

To determine if the developmental gradient has a confounding impact on the inhibitor effects, multivariate analyses were performed for a given age of plants (6 or 10-day old) and as a function of endosidin-7 concentration. The criteria for significance were defined by relative changes in the leaves or roots, in 6-day old plants (two concentrations of endosidin-7; 3 or 5 μM) or 10-day old plants (three concentrations of endosidin-7; 3, 5, or 10 μM) to the plants of the same growth stage without treatment control. For conditions (FC > 1.5 and p-value < 0.05), the analysis identified three metabolites (dimethylamine, glycerone and syringate), and with a reduced p-value to 0.1 identified six additional metabolites. Taking together, these multivariate analysis results unequivocally demonstrate that the metabolic differences between samples are primarily caused by treatment with different endosidin-7 concentrations rather than the developmental gradient of the sample.

### Endosidin-7 induces changes in the primary arabidopsis metabolism

After verifying that endosidin-7 treatment caused significant changes in the metabolome composition, surpassing that of the developmental gradient, we focused on identifying the most prominently altered metabolites. In order to increase the metabolite detection sensitivity within the biomarker window and the statistical power of biological replicates, we focused on comparing the endosidin-7 effect between treated and untreated samples in the leaves or roots, independent of the developmental stage. Metabolite changes were considered significant when a threshold of fold-change (log2) > 1.5 with a corresponding adjusted *p*-value < 0.05 was observed. A larger number of metabolites (> 100) was identified by Chemonex, however relevant plant metabolites were only considered if they can be found in the spectra of all endosidin-7 treated samples versus the control samples. The presence of fifty-three metabolites was significantly changed upon endosidin-7 treatment in leaves or roots (*p* < 0.05), as listed in the **S1 Table** (endosidin-7 treatment n = 15, control n = 12).

**Fig 3:**
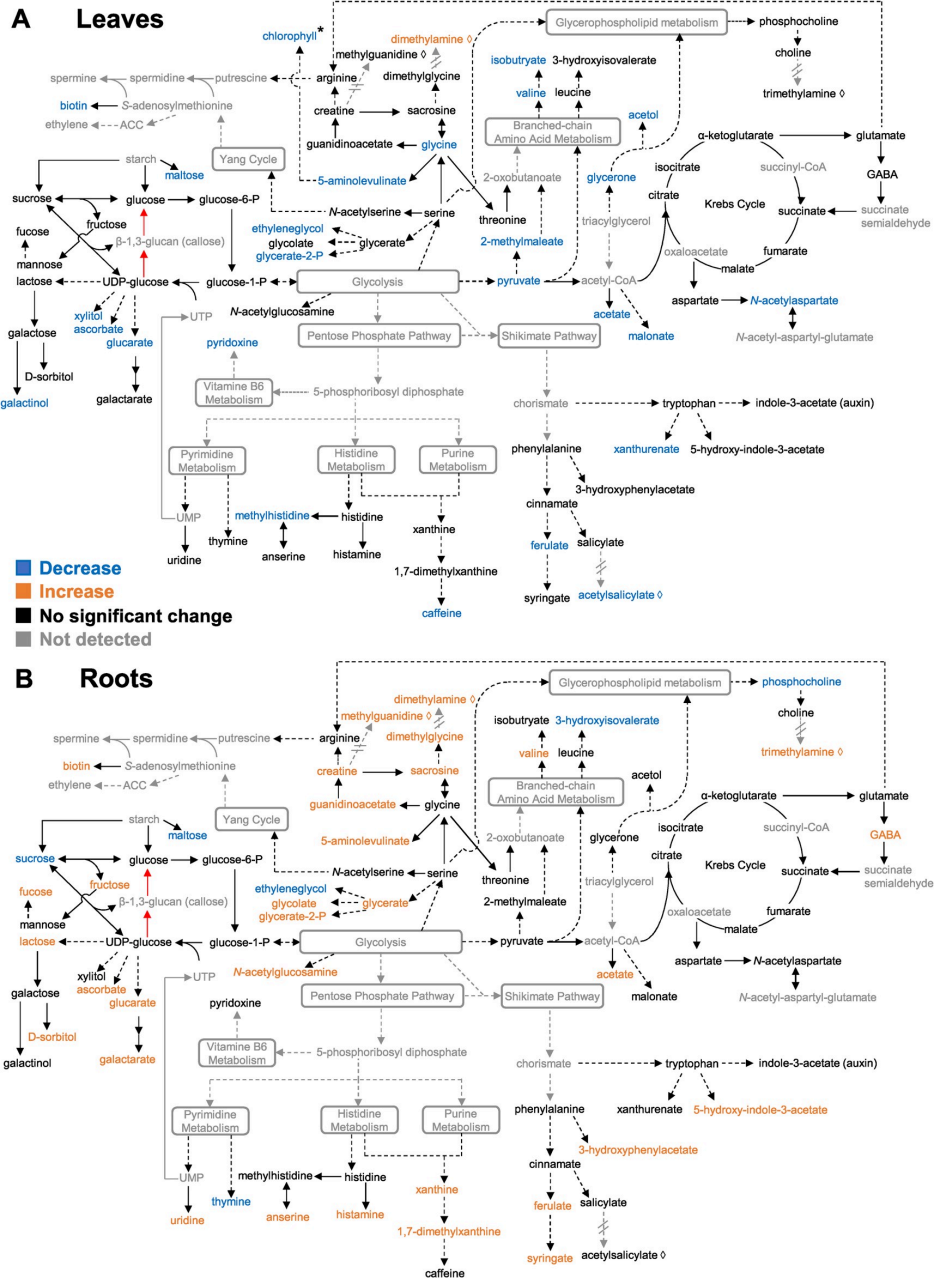
Metabolic pathway map of altered metabolites in (A) leaves and (B) roots of arabidopsis seedlings upon endosidin-7 treatment. Backbone pathways are adapted from KEGG (genome.jp/kegg). Each solid arrow indicates one enzymatic step, and each dashed arrow indicates multiple enzymatic steps. Red arrows represent callose synthase and β-1,3-glucanase pathways, respectively. Grey rounded rectangles denote major pathways with multiple steps. Compounds in grey were not detected in the analysis, while dashed arrows with double cross lines indicate metabolic steps not previously reported in plants. Compounds in orange exhibited a significant increase; compounds in blue indicate a significant decrease, and compounds in black indicate no significant change upon endosidin-7 treatment (significant criteria *p* < 0.05 in multivariate analysis, with exceptions and details indicated in S1 Table). Chlorophyll change is inferred from the analysis of Fig 1C and denoted by *.

**Fig 4:**
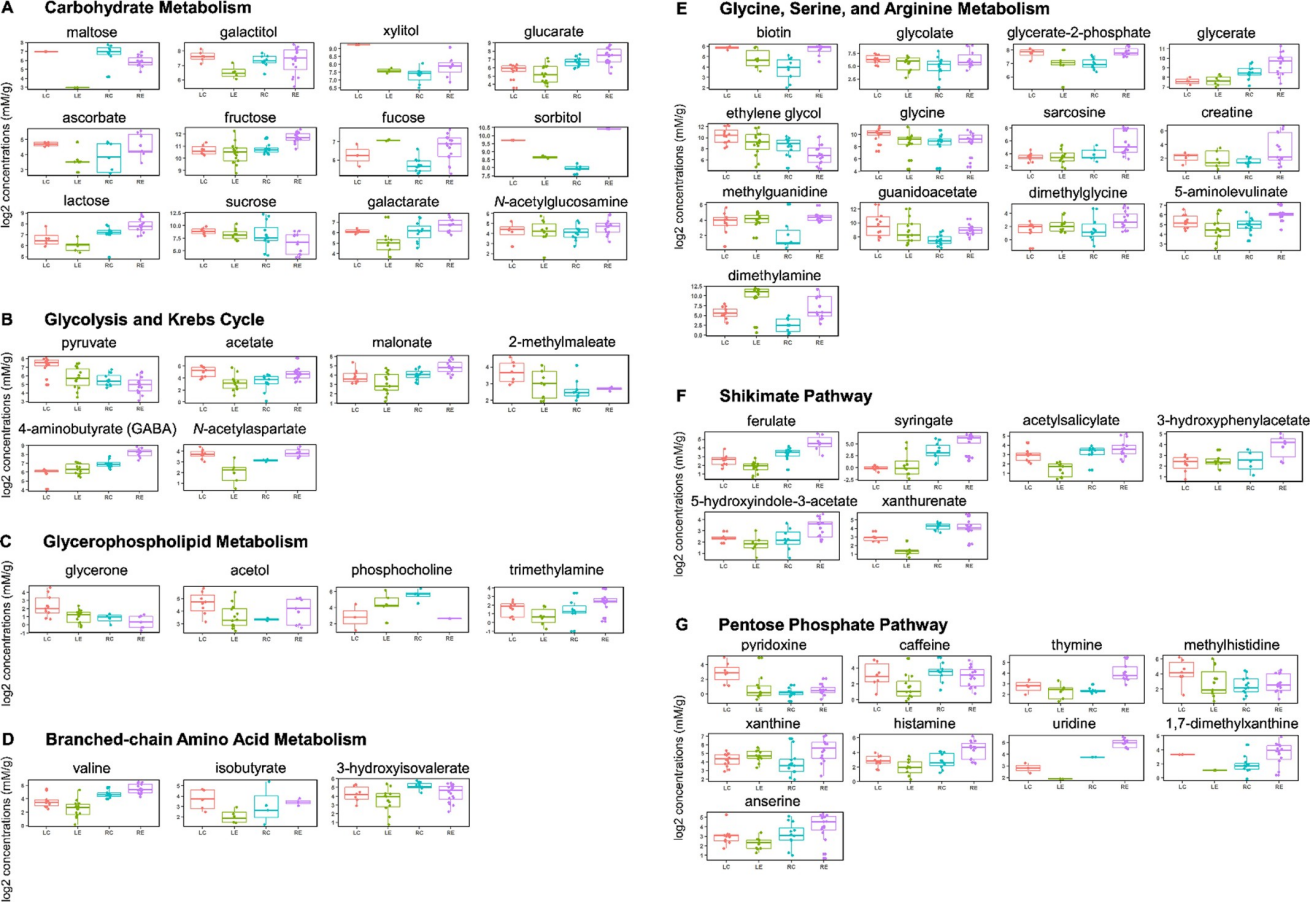
Differential levels of NMR detected metabolites in roots and leaves under endosidin-7. Metabolites were categorized according to their involvement in major or upstream pathways. Each panel lists a specific metabolite for LC (Control leaves, n = 12), LE (endosidin-7 treated leaves, n = 15), RC (Control roots, n = 12) and RE (endosidin-7 treated root, n = 15). Concentrations are expressed in mM/g (log2) with reference to the internal standard TSP.

Individual compounds, shown in **S1 Table,** were first explored for their roles in arabidopsis metabolic pathways using the Kyoto Encyclopedia of Genes and Genomes (KEGG, genome.jp/kegg, [39]). Their putative involvement and role in the biochemical pathways of leaves and roots are summarized in a network map adapted from KEGG pathways, **Fig 3**. Detected metabolites can be categorized into components and derivatives of seven major metabolic pathways, including carbohydrate metabolism [40], glycolysis and Krebs cycle [41, 42], glycerophospholipid metabolism, branched-chain amino acid metabolism [43], glycine, serine, and arginine metabolism [44, 45], shikimate pathway [46], and the pentose phosphate pathway [47], **S1 Table**. The average concentrations of the metabolites in the control or endosidin-7 treatment are listed for leaves and roots (**S2 Table**). Individual biological replicates are shown in the boxplot to indicate the variation of each of the metabolites in the treatment (**Fig 4**). Most of the detected metabolites are regarded as part of primary metabolism (**Fig 4A–4G**) and are involved in central plant growth and developmental processes.

Four metabolites with uncharacterized biosynthetic pathways were identified: methylguanidine, dimethylamine, trimethylamine, and acetylsalicylate. These metabolites were plotted in the metabolite map based on structural similarity with putative precursors, indicated by lozenge symbol (◊), and linked with putative precursors by dashed lines with a break in the arrows (**Fig 3)**. Methylguanidine and trimethylamine have been independently reported in plant metabolomes using NMR spectroscopy [48] and liquid chromatography-tandem mass spectrometry [49].

The levels of the 53 compounds (**S1 Table**) are also shown across the different developmental stages in untreated samples and are listed in the **S3 Table**. According to the aforementioned multivariate analysis, none of these compounds showed significant changes across the observed developmental gradient in either roots or leaves. Apart from primary metabolism compounds, there were very few specialized metabolites detected, likely because they are generally of low abundance in arabidopsis and thus challenging to identify by 1D NMR profiling.

The dominant part of the modulated root metabolites showed an increase upon endosidin-7 treatment (**Fig 3B**), contrasting a decrease in leaves (**Fig 3A**). This likely reflects the difference in metabolic needs and compensatory mechanisms in aerial tissues and roots. The levels of most reduced sugars and their derivatives showed increased accumulation in roots and reduced leaves upon endosidin-7 treatment. Maltose and sucrose were exceptions, showing a decreased accumulation in roots (**Fig 4A** and **S2 Table**). A physiological concentration of maltose is known to maintain membrane potential and protect the photosynthetic electron transport chain *in vitro* [50]. The decrease of maltose in both roots and leaves upon endosidin-7 treatment may indicate a disrupted primary metabolism. In roots, endosidin-7 induced an increase of compounds upstream of the polyamine biosynthesis, including creatine, guanidinoacetate, and sarcosine (**Fig 4E**), which are derivatives of glycine, serine, and arginine. In addition, endosidin-7 treated roots exhibited an increase in 4-aminobutyrate (GABA) (**Fig 4B**), a Krebs cycle derivative., whose production is closely related to *in vivo* polyamine levels under stress [51, 52]. Endosidin-7 also modulated levels of ferulate, syringate, 5-hydroxyindole-3-acetate, and xanthurenate in both roots and leaves (**Fig 4F**). As products of the shikimate pathway, these compounds are phenylpropanoid and tryptophan derivatives related to the precursors for the biosynthesis of plant hormones, including auxin and salicylic acid [53, 54]. Taken together, these metabolite changes suggest a pronounced effect of endosidin-7 on plant hormone biosynthesis pathways.

### The pool of UDP-glucose and glucose are not significantly affected by endosidin-7

We did not detect significant changes in the direct metabolic substrate and degradation product of callose (β-1,3-glucan), namely UDP-glucose and glucose, upon endosidin-7 treatment (**Fig 3**). This shows that inhibition of cytokinesis-specific callose deposition does not cause a global change in the precursor pool. Given that UDP-glucose is involved in many metabolic activities and various cell wall polysaccharide biosynthetic steps, such as starch and cellulose, the modulation of the transient accumulation of callose at the cell plate might not be discernable.

**Fig 5:**
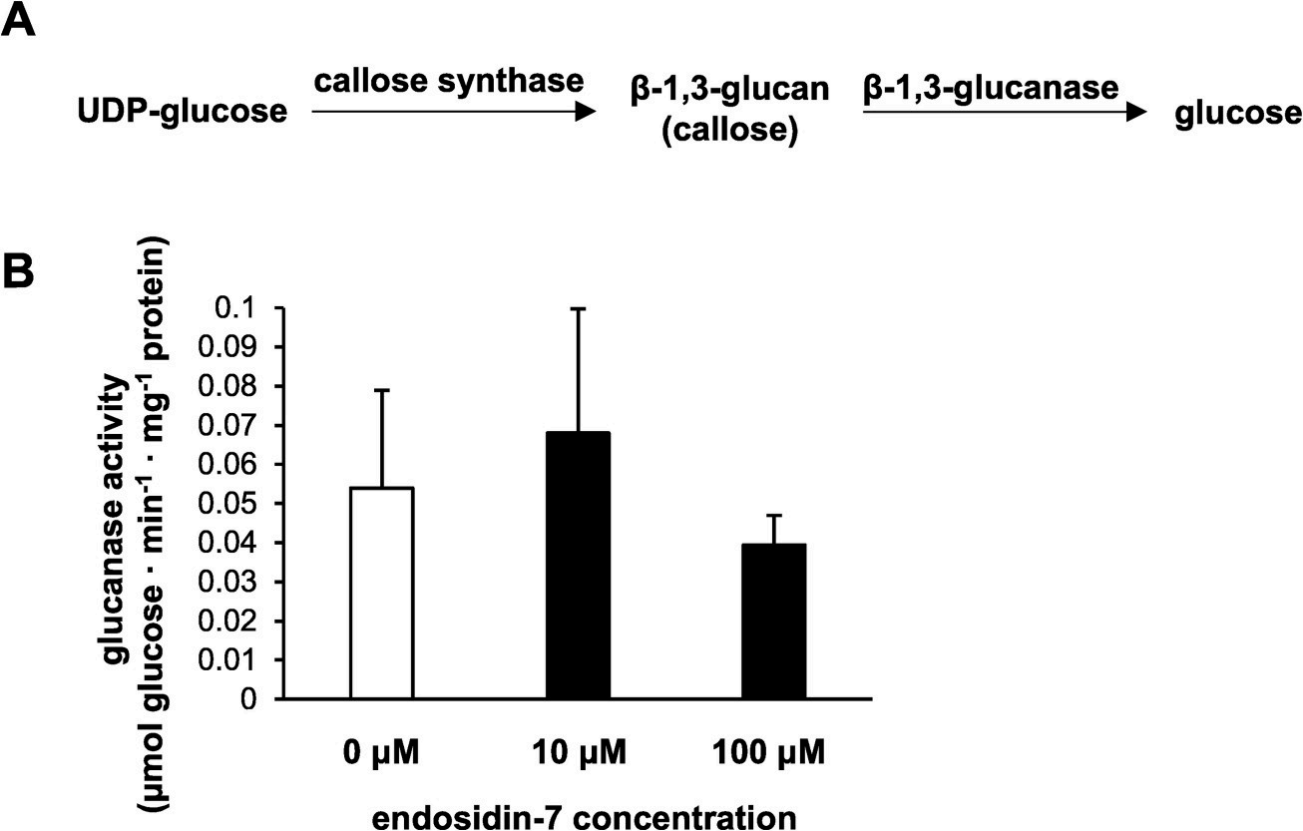
Endosidin-7 treatment does not affect glucanase activity. (A) Callose synthase and β-1,3-glucanase regulate the dynamic equilibrium of the callose deposition *in vivo*. (B) Glucanase activity in crude extracts of arabidopsis seedling leaves. Activities are measured via the amount of glucose generated by the incubation of desalted crude extracts with laminarin. No statistical difference was observed on the glucanase activity between the three treatments of DMSO, 10 μM endosidin-7, or 100 μM endosidin-7. Data are presenting the mean ± SD (n = 4).

Callose deposition is regulated by the activity of both callose synthases and β-1,3-glucanases (**Fig 5A)** [55]. Arabidopsis contains twelve homologs of callose synthases [56] and fifty homologs of β-1,3-glucanases [57]. It is plausible that endosidin-7 enhances the activity of cytokinetic β-1,3-glucanase(s), leading to higher phragmoplast callose degradation, thereby constraining polymer availability. To test this hypothesis, we examined the total glucanase activity modulation in arabidopsis crude extracts upon treatment with endosidin-7. At 10 μM and 100 μM endosidin-7, total glucanase activity did not show a statistically significant change compared to the DMSO control (**Fig 5B**). This strongly suggests that global β-1,3-glucanase activity is not affected by endosidin-7, corroborating our NMR observations that endosidin-7 does not cause a significant change in the direct metabolic substrate and degradation product of callose.

## Discussion

### NMR-based investigation reveals small molecule induced metabolomics changes

Plant metabolomics can be defined as the quantitative measurement of the time-related multiparametric metabolic response of plants to environmental stimuli or genetic modification. The metabolomic information content complements the genomic and proteomic approaches toward the interpretation of biological mechanisms and their function [58, 59]. Transcriptome and proteome analyses have been extensively recruited to study how organ-specific responses are coordinated during growth and are assisting a better understanding of plant development [60–64]. Considerable strides have been made in recent analytical and methodological advances, especially in the field of gene expression analysis, that now allow single-cell level-based studies. These, in turn, can unravel how gene networks are organized and regulated at the cellular level [65–67].

In the field of metabolomics, attempts to describe the metabolome of single cells have been based on tandem mass spectrometry [68]. In pioneering, spatial resolution improving work, a survey of the subcellular distribution of metabolites in cytosolic, vacuolar, and plastid fractions of arabidopsis leaves was performed by Krueger and colleagues using GC-TOF/MS and LC/MS. The results provided a topological metabolite map and took initial steps toward analyzing the metabolic dynamics between subcellular compartments [69].

While a metabolomic analysis can be performed using several mass spectrometry-based analytical chemistry techniques, NMR spectroscopy-based metabolomics analysis has some distinct advantages [70, 71]. These include easy and rapid sample preparation, elimination of derivatization analysis, which in turn allows high-throughput and quantitative analysis with a single internal standard [72–74]. In plant biology, NMR techniques have been utilized mostly for the characterization of cell wall polymers [17, 75–77]. However, the most current applications of NMR-based metabolomics in plants emphasize analytical data accumulation and sample classification, not developing 1H NMR spectroscopy as a tool to study metabolic networks [78]. This bias might be attributable to the limited availability of open-access NMR spectral libraries for plant metabolites [79, 80]. NMR studies on the whole plants have been conducted to evaluate the response of the plant defense inducer benzothiadiazole [81], the hormone methyl jasmonate involved in various signaling pathways, including tissue wound response [27], the trafficking inhibitor Sortin 1 [25], and the fungal pathogen *Verticillium dahliae* [82]. The arabidopsis metabolic profiles, with and without pharmacological inhibition, presented here can contribute to an enhanced understanding of general plant responses to small-molecule stimuli.

### Leaf and root metabolome modulations to dissect small molecule induced plant responses

We analyzed, through ^1^H NMR spectroscopy profiling, arabidopsis leaf and root metabolites responding to the small molecule endosidin-7. Leaves and roots reacted distinctly different, clearly demonstrating organ-specific responses to endosidin-7, which could be quantified via multivariate analysis (**Fig 4 and S2 Table**). Metabolomics has been utilized earlier to study plant abiotic or biotic stress [83, 84]. Discriminating root and leaf metabolic responses were investigated through GC or LC-MS upon sublethal cadmium exposure and high salt and low potassium stress. Principal component analysis (PCA) in these three independent metabolomics studies consistently indicated differential responses in root and leaf tissue of arabidopsis and barley [85–87]. In addition to the studies mentioned above, Novák et al. studied the organ-specific auxin metabolome of both roots and leaves of wild type and auxin over-producing arabidopsis lines by LC-MRM-MS [88]. PCA analysis revealed that the overproduction of auxin leads to distinct metabolome modulations, in which up- or down-regulation of the metabolites in leaves and roots does not follow a synchronized pattern [88]. Fontaine and colleagues showed in an NMR metabolomics study, using PCA on leaves, stems, and roots of *GLUTAMATE DEHYDROGENASE 3* mutants and wild type arabidopsis, that organ-specific metabolomics responses are taking place. These changes include amino acids, organic acids, and sugars [89]. In mold-resistant melon rootstocks (roots), and susceptible watermelon scions (aerial parts), organ-specific metabolite changes were observed via NMR upon exposure to the powdery mildew disease. Notably, the concentrations of root and leaf metabolites changed in opposite directions. The authors put forward the hypothesis that translocation of metabolites between rootstocks and scions through the vascular system is responsible for the antiparallel metabolome modulation [90].

Our observed trend of metabolite level changes in leaves and roots upon endosidin-7 treatment is also strikingly different (**Fig 3**and **S1 Table**), despite the similarity in cytokinesis arrest at the cellular level [2]. One plausible explanation of this antiparallel root and leave response is the aberrant translocation of metabolites between plant organs under endosidin-7 treatment. The levels of ferulate and syringate, both precursors of lignin biosynthesis, are affected upon endosidin-7 treatment (**Fig 3**and **S1 Table**). Aberrant lignin biosynthesis may further affect xylem development in arabidopsis seedlings [91], which in turn interferes with nutrient translocation and potentially giving rise to the antiparallel metabolome modulation in roots and leaves. Another equally plausible explanation is that intrinsic organ-specific regulation of primary metabolic pathways is not concomitant in plant organs under chemically induced stressed conditions. The cited studies above, together with our presented data, illustrate the importance of organ-specific investigations to assess the responses of plants comprehensively via NMR metabolomics.

### Long term endosidin-7 treatment may induce hormonal responses in arabidopsis

Endosidin-7 inhibits specifically cytokinetic callose deposition but does not affect wounding stress-induced callose deposition or its deposition at sieve cells [2]. Given the specificity of endosidin-7 on cytokinetic callose, we performed a long-term endosidin-7 treatment for 6 and 10 days to investigate the metabolic phenotype that captures both the long-term growth inhibition and the cellular phenotype of arrested cell division. Together, the morphological phenotype of reduced growth, altered gravitropic response, and metabolite changes induced by endosidin-7 treatment suggest a hormonal response from endosidin-7 exposure. Loss of gravitropism is a sign of possible hormonal regulation disruption, as it is regulated by crosstalk between auxin and other hormones [92, 93]. We did not observe a significant change in indole-3-acetate (auxin) levels upon endosidin-7 treatment; however, 5-hydroxyindole-3-acetate showed a significant increase in roots (**Fig 4F**). In independent plant metabolomics studies in melon, soybean, and *Isatis indigotica* [90, 94, 95], 5-hydroxyindole-3-acetate is reported to be putatively synthesized from tryptophan and it is speculated to affect auxin metabolism in plants [90, 95]. Considering the increase of xanthine and 1,7-dimethylxanthine in roots (**Figs 3 and 4G**), it is likely that downstream of the purine metabolism, cytokinins synthesized from the deoxyxylulose pathway [96, 97] are affected upon endosidin-7 treatment. The plant hormone salicylate, which is involved in both abiotic and biotic stress [98, 99], was not significantly changed, but levels of acetylsalicylate showed a 60% reduction upon endosidin-7 treatment in leaves (**Fig 4F and S2 Table**). Ambiguity for the identification of salicylate and acetylsalicylate in the present study is possible due to the strong similarity in the aromatic spectral region (in the range of 7–9 ppm) of these molecules. Further, components of the polyamine biosynthesis pathways are affected upon endosidin-7 treatment. Altogether, our data strongly suggest that the imbalance of certain hormone levels during prolonged treatment of endosidin-7 could lead to the induction of the global metabolite changes.

The proposed hormonal regulation is supported by previous long-term hormone treatment omics studies [100, 101]. Earlier microarray analysis revealed that 35 primary metabolism-related genes, involved in light signaling, nutrient uptake, and photosynthesis were altered in arabidopsis shoots treated with 5 μM isopentenyladenine (a synthetic cytokinin) for 4 days [100, 101]. Similarly, in another long-term cytokinin triggered study, lettuce treated by benzylaminopurine or meta-topolin for 13 days showed reduced accumulation of photosynthetic pigments and inhibition of photosystem II activity [102]. The phenotypes of endosidin-7 treated seedlings, plus the plethora of metabolite changes related to the primary metabolism (**Fig 3**) accompanying the loss of chlorophyll (**Fig 1C**), also suggest an aberrant hormonal regulation, as chlorophyll synthesis is tightly regulated by the balance of auxin and cytokinin [103, 104]. Both auxin and cytokinin are responsible for the initiation of the G1/S phase transition prior to cell division, a prerequisite for cell division through the regulation of cyclin-dependent kinases [105–108]. Further, crosstalk exists between the auxin and cytokinin biosynthetic pathways via the direct regulation of biosynthesis genes and transporters [109]. Endosidin-7 induced metabolome changes could reflect a compensatory mechanism counteracting the inhibition of cytokinesis and plant growth resulting from a cytokinin and auxin imbalance.

The effect of endosidin-7 inhibition on callose deposition at the division plate can be observed after a short two hours of pulse treatment [2]. This time frame is in line with characteristic hormonal responses, where gene expression or metabolite level changes are usually observed after a few hours of cytokinin or auxin induction [110–113]. Given that the cellular phenotype of cell plate disruption is observed after only two hours when treated with 50 μM, endosidin-7, these conditions could be used in future NMR metabolomic studies to dissect the long-term metabolite effects from the short-term.

### Summary and perspectives

We performed an organ-specific NMR-based metabolomics study of arabidopsis leaves and roots at different developmental stages and treatments with various concentrations of the specific cytokinesis inhibitor endosidin-7. Metabolome analyses indicated that cytokinesis inhibition by endosidin-7 likely disrupts primary metabolism and hormonal regulation. This study provides metabolomics references for early stages of arabidopsis development, indicates multiple metabolic pathways affected by endosidin-7, and highlights the relevance of organ or tissue-specific investigation in plants for an accurate and comprehensive assessment of plant metabolome modulations.

Our organ-specific examination highlighted the importance of spatial resolution in metabolite analysis. Given the complex interactions between metabolic pathways, future studies allowing higher spatial and temporal resolutions are essential for unmasking the different layers of interaction, particularly upon exogenous stimuli. There are various ways to envision how this complex task could be efficiently addressed through future developments, two promising ones are: a) automation through partially robotic extraction of the required substantial amounts of tissue, and b) improving the sensitivity of metabolite identification and metabolic flux analysis to reduce the required sample volumes. The use of stable isotopic enriched and multidimensional NMR metabolomics might be key for the latter [71, 114–116]. Technological advances, in combination with cell synchronization, might ultimately uncover very delicate metabolite changes during various cellular processes, including cytokinesis.

## Supporting information

S1 Fig Representative NMR spectra of roots and leaves (10-day old plants), and simulated spectra based on the metabolites that are significantly alerted due to endosidin-7 treatment. (PDF)

S2 Fig Statistical analyses of the arabidopsis metabolome without endosidin-7 treatment. (A) PLS-DA analysis of leaves and root metabolomes. (B) Correlation analysis among 4, 5, 6, and 10-day old samples without endosidin-7 treatment. Triplicates of each developmental stage, ranked with the highest correlation with each other, are highlighted in a dashed square. Values in the squares represent the correlation coefficient between every two samples in the plot. The histogram shows the density estimation. The scatter plot displays the strength of the relationship. (C) PLS-DA analysis of metabolomics data of arabidopsis seedlings at different days after germination. Ellipses represent a 95% confidence region of the classification.
(PDF)

S3 Fig Evaluation of the PLS-DA model. A 10-fold cross-validation, with three different measures, was performed. Blue bars indicate the accuracy of the model, pink bars (R^2^, variations) indicate the goodness of fit, and light-blue bars (Q^2^, prediction of the model) indicate the goodness of prediction. Good predictions with a high Q^2^ value are marked by *. PLS-DA correspond to the figures: Figs 2A and 2B and S2C.
(PDF)

S4 Fig PCA analysis of the data corresponding to PLS-DA analysis shown in Figs 2A and 2B and S2C. As indicated above, S2C Fig indicates analysis of metabolomics data of arabidopsis seedlings at different days after germination without chemical treatment, while Fig 2A describes PLS-DA analysis of all NMR data under different endosidin-7 concentrations. Fig 2B shows PLS-DA analysis of only 10-day old plants for different endosidin-7 concentrations. Score plots with the respective variances are shown in parenthesis.
(PDF)

S1 Table Significantly changed leaves and root metabolites upon endosidin-7 treatment. The difference of metabolite levels in arabidopsis seedlings for endosidin-7 treatment (n = 15) versus the control (n = 12) is expressed by log2 fold change. The length of the colored bar is proportional to the value, with decreases in blue and increases indicated in orange. A threshold of 1.5 (log2) was used in the multivariate analysis to calculate the *p*-value of significance.
(PDF)

S2 Table Quantification of metabolite level changes upon endosidin-7 treatment in leaves and roots. Concentrations (mean±SD) are expressed in mM/g (log2) with reference to the internal standard TSP (n = 12 for controls, n = 15 for endosidin-7 treated).
(PDF)

S3 Table Quantification of metabolite level modulations in leaves and roots during seedling development. Without endosidin-7 treatment, multivariate analysis does not show significant concentration modulations for these compounds (threshold of 1.5 (log2), *p* < 0.05). Concentrations (mean±SD) are expressed in mM/g (log2) with reference to the internal standard TSP (n = 3 for each developmental stage). N.D. denotes “not detected”.
(PDF)
