# Supplementary material for: NMR spectroscopy analysis reveals differential metabolic responses in arabidopsis roots and leaves treated with a cytokinesis inhibitor
Source: PLoS One. 2020 Nov 6;15(11):e0241627. doi: 10.1371/journal.pone.0241627 (PMC7647083; doi:10.1371/journal.pone.0241627)
Supplement: S1 Fig — (PDF) [file pone.0241627.s001.pdf]

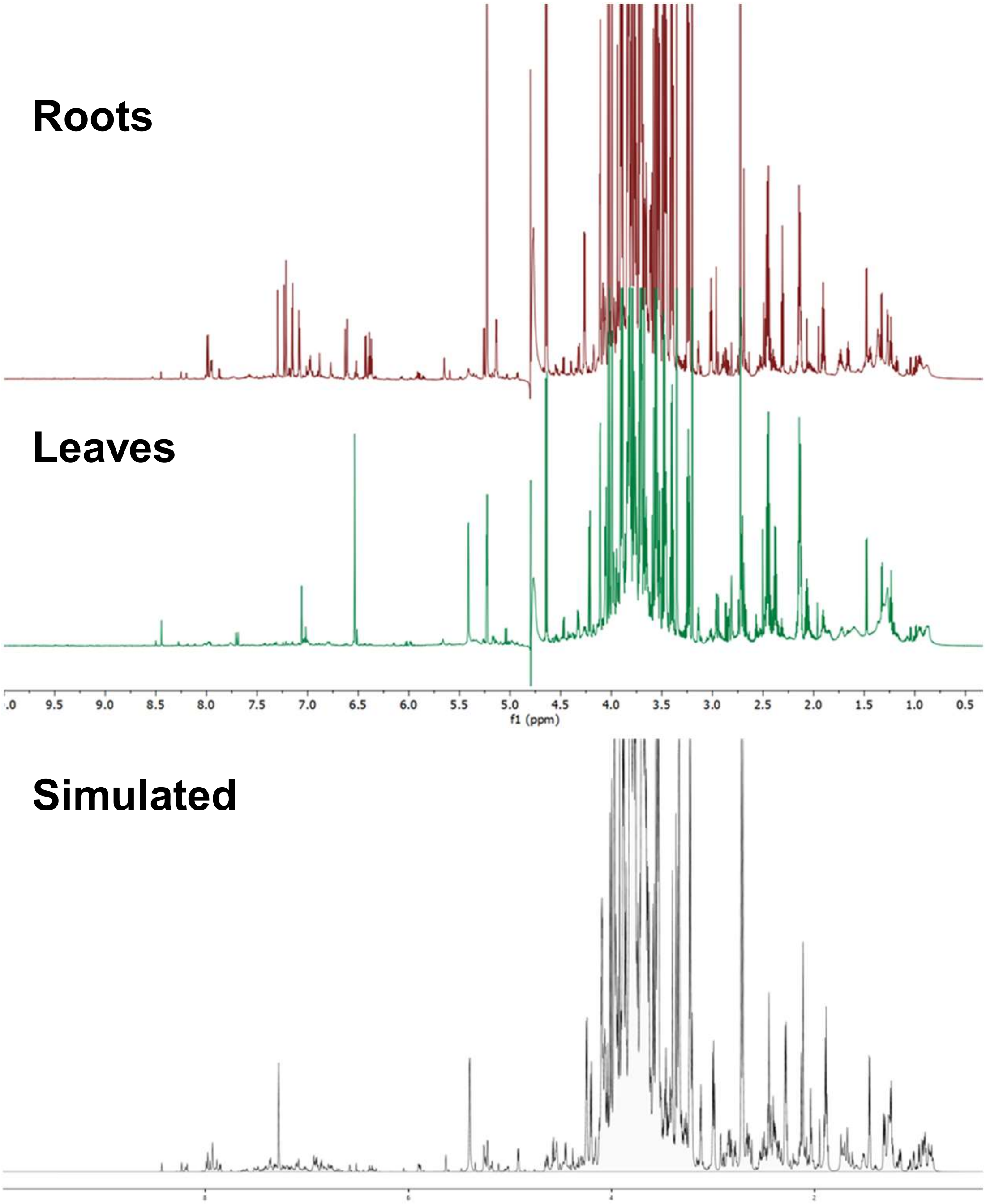

S1 Fig. Representative NMR spectra of roots, leaves (10-day old plants), and simulated spectra based on the metabolites that are significantly alerted due to endosidin-7 treatment.
