# Supplementary material for: NMR spectroscopy analysis reveals differential metabolic responses in arabidopsis roots and leaves treated with a cytokinesis inhibitor
Source: PLoS One. 2020 Nov 6;15(11):e0241627. doi: 10.1371/journal.pone.0241627 (PMC7647083; doi:10.1371/journal.pone.0241627)

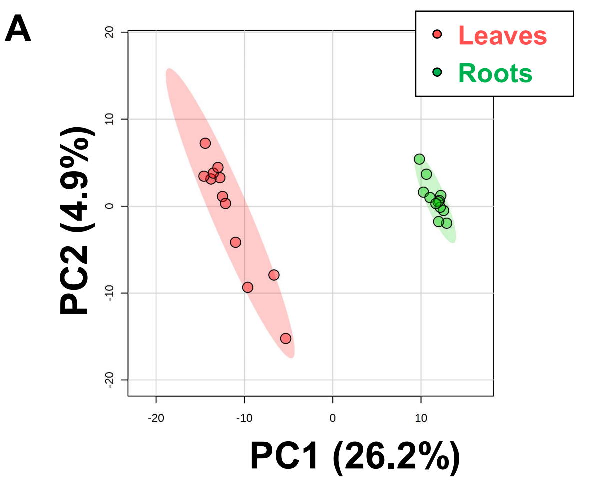

S2 Fig. Statistical analyses of arabidopsis metabolome without endosidin-7 treatment.
(A) PLS-DA analysis of leaves and roots metabolome. (B) Correlation analysis among 4, 5, 6, and 10-day old samples without endosidin-7 treatment. Triplicates of each developmental stage were ranked with the highest correlation with each other, highlighted in a dashed square. Between every two samples in the plot, values in the square represent the correlation coefficient, histogram indicates density estimation, and scatter plot displays the strength of the relationship. **(C) PLS-DA analysis of metabolomics data of arabidopsis seedlings at different days after germination.** Ellipses represent a 95% confidence region of the classification.

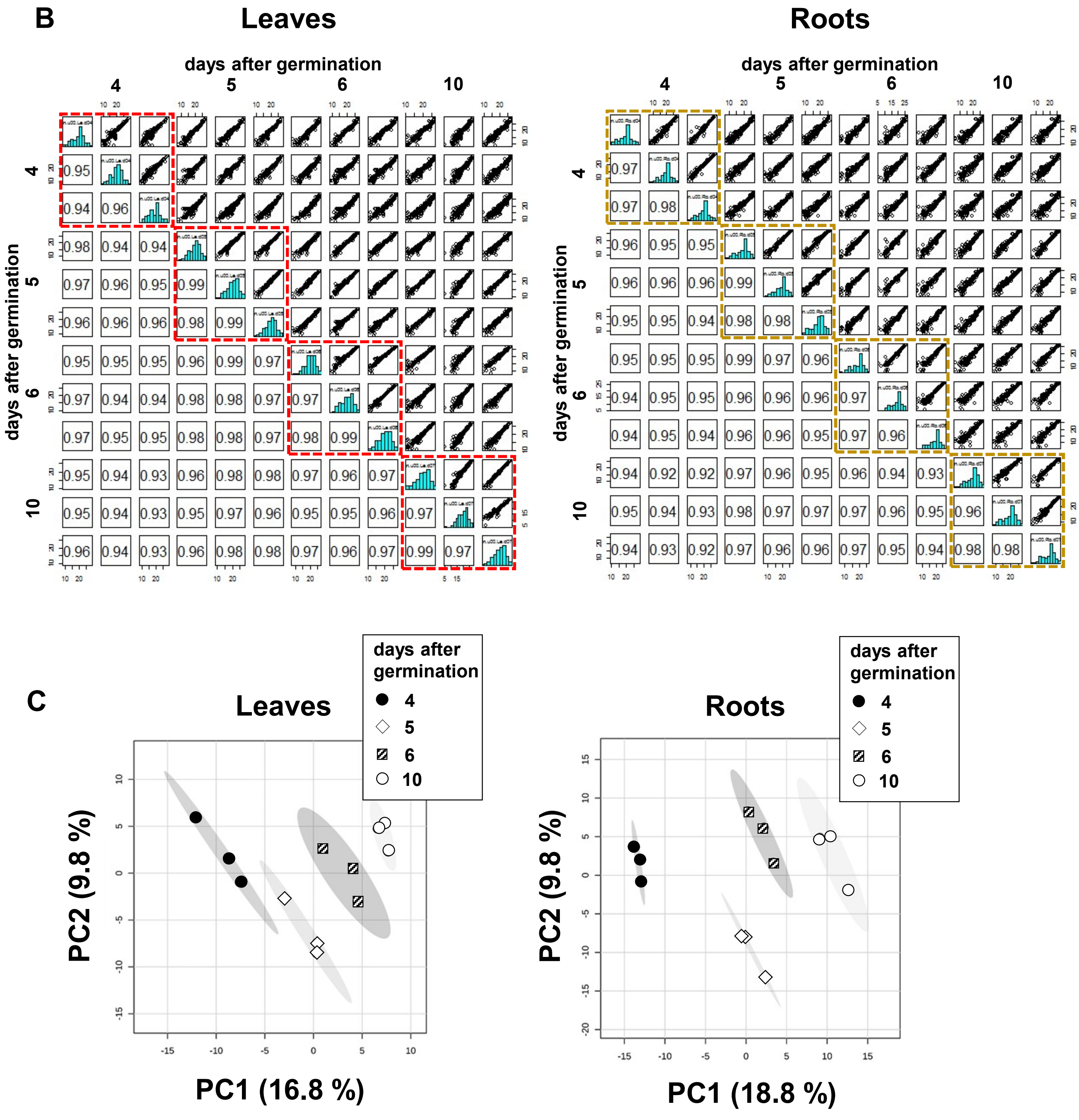

Supplement: S2 Fig — (A) PLS-DA analysis of leaves and root metabolomes. (B) Correlation analysis among 4, 5, 6, and 10-day old samples without endosidin-7 treatment. Triplicates of each developmental stage, ranked with the highest correlation with each other, are highlighted in a dashed square. Values in the squares represent the correlation coefficient between every two samples in the plot. The histogram shows the density estimation. The scatter plot displays the strength of the relationship. (C) PLS-DA analysis of metabolomics data of arabidopsis seedlings at different days after germination. Ellipses represent a 95% confidence region of the classification. (PDF) [file pone.0241627.s002.pdf]
