# Supplementary material for: NMR spectroscopy analysis reveals differential metabolic responses in arabidopsis roots and leaves treated with a cytokinesis inhibitor
Source: PLoS One. 2020 Nov 6;15(11):e0241627. doi: 10.1371/journal.pone.0241627 (PMC7647083; doi:10.1371/journal.pone.0241627)
Supplement: S3 Fig — A 10-fold cross-validation, with three different measures, was performed. Blue bars indicate the accuracy of the model, pink bars (R2, variations) indicate the goodness of fit, and light-blue bars (Q2, prediction of the model) indicate the goodness of prediction. Good predictions with a high Q2 value are marked by *. PLS-DA correspond to the figures: Figs 2A and 2B and S2C. (PDF) [file pone.0241627.s003.pdf]

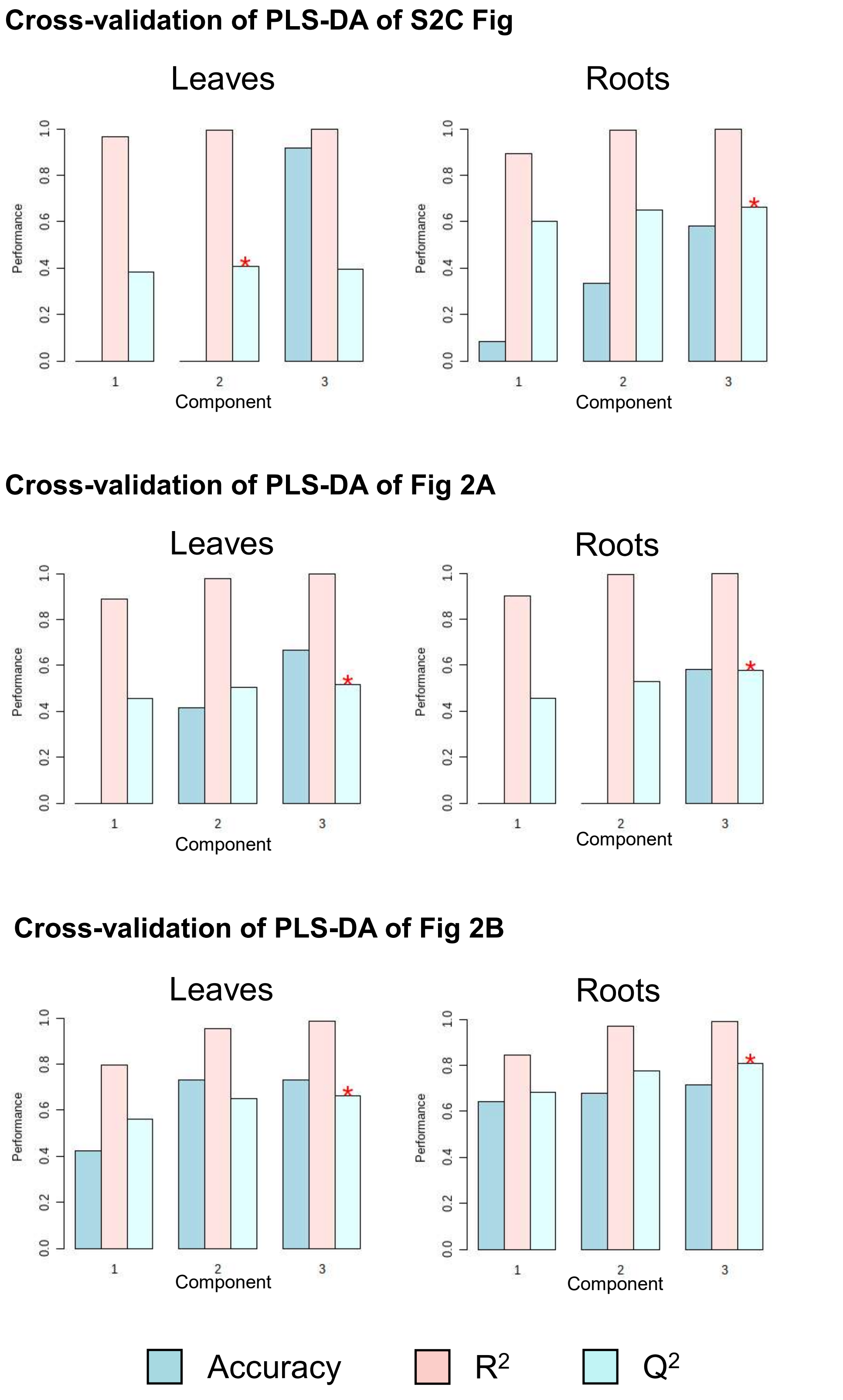

S3 Fig. Evaluation of the PLS-DA model.
A 10-fold cross-validation, with three different measures, was performed. Blue bars indicate the accuracy of the model, pink bars (R2, variations) indicate the goodness of fit, and light-blue bars (Q2, prediction of the model) indicate the goodness of prediction. Good predictions with a high Q2 value are marked by *. PLS-DA correspond to the figures: **S2C Fig**, **Fig 2A**, and **Fig 2B**.
