# Supplementary material for: NMR spectroscopy analysis reveals differential metabolic responses in arabidopsis roots and leaves treated with a cytokinesis inhibitor
Source: PLoS One. 2020 Nov 6;15(11):e0241627. doi: 10.1371/journal.pone.0241627 (PMC7647083; doi:10.1371/journal.pone.0241627)
Supplement: S4 Fig — As indicated above, S2C Fig indicates analysis of metabolomics data of arabidopsis seedlings at different days after germination without chemical treatment, while Fig 2A describes PLS-DA analysis of all NMR data under different endosidin-7 concentrations. Fig 2B shows PLS-DA analysis of only 10-day old plants for different endosidin-7 concentrations. Score plots with the respective variances are shown in parenthesis. (PDF) [file pone.0241627.s004.pdf]

### PCA analysis of the samples in S2C Fig

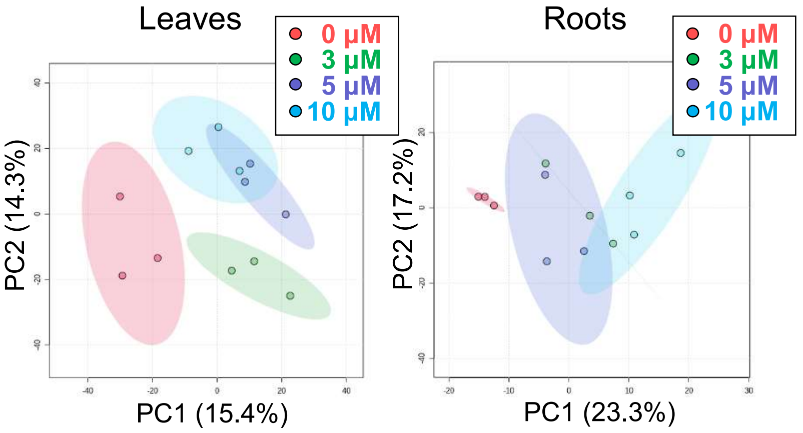


### PCA analysis of the samples in Fig 2A

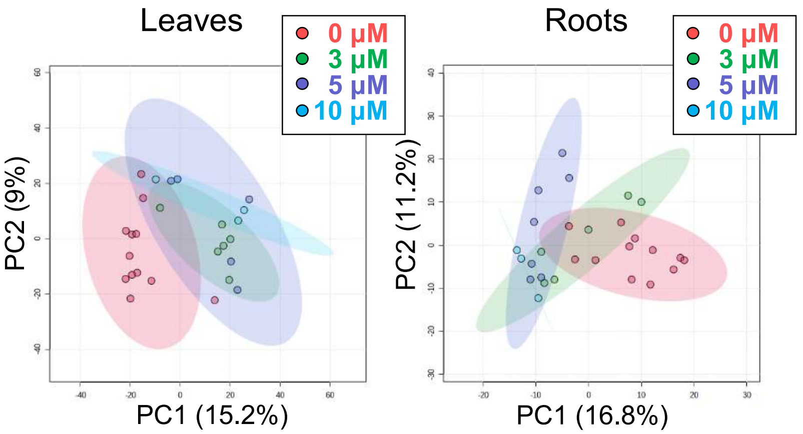


### PCA analysis of the samples in Fig 2B

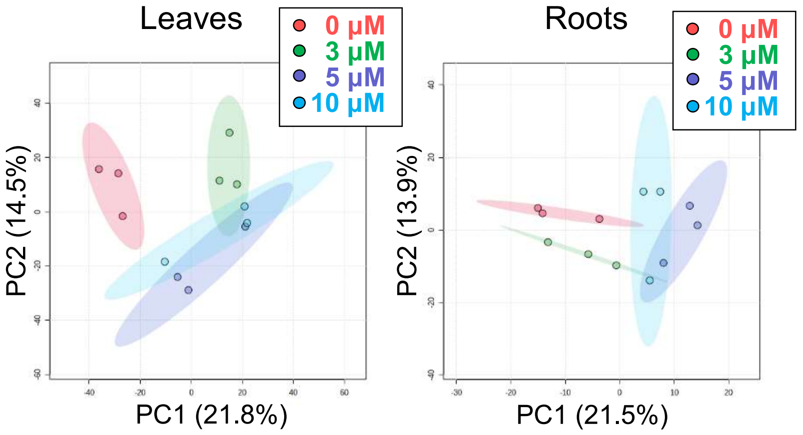


**S4 Fig. PCA analysis of the data corresponding to PLS-DA analysis shown in S2C Fig and Fig 2A and Fig 2B.**
As indicated above, **S2C Fig** indicates analysis of metabolomics data of arabidopsis seedlings at different days after germination without chemical treatment, while **Fig 2A** describes PLS-DA analysis of all NMR data under different endosidin-7 concentrations. **Fig 2B** shows PLS-DA analysis of only 10-day old plants for different endosidin-7 concentrations. Score plots with the respective variances are shown in parenthesis.
