# Supplementary material for: NMR spectroscopy analysis reveals differential metabolic responses in arabidopsis roots and leaves treated with a cytokinesis inhibitor
Source: PLoS One. 2020 Nov 6;15(11):e0241627. doi: 10.1371/journal.pone.0241627 (PMC7647083; doi:10.1371/journal.pone.0241627)
Supplement: S1 Table — The difference of metabolite levels in arabidopsis seedlings for endosidin-7 treatment (n = 15) versus the control (n = 12) is expressed by log2 fold change. The length of the colored bar is proportional to the value, with decreases in blue and increases indicated in orange. A threshold of 1.5 (log2) was used in the multivariate analysis to calculate the p-value of significance. (PDF) [file pone.0241627.s005.pdf]

	Leaves		Roots	
	log2 FC (ES7 - Control)	*p* value	log2 FC (ES7 - Control)	*p* value
**Carbohydrate Metabolism**				
maltose	-4.01	0.00	-0.88	0.02
galactitol	-1.09	0.03	0.02	0.94
xylitol	-1.65	0.06	0.56	0.14
glucarate	-1.10	0.04	0.64	0.09
ascorbate	-1.05	0.07	0.81	0.09
fructose	-0.27	0.38	0.92	0.00
fucose	0.82	0.21	0.94	0.00
sorbitol	-1.05	0.17	2.47	0.00
lactose	-0.65	0.17	0.75	0.01
sucrose	-0.63	0.52	-1.78	0.03
galactarate	-0.36	0.35	0.70	0.06
N-acetylglucosamine	0.03	0.95	0.60	0.09
**Glycolysis and Krebs Cycle Derivatives**				
pyruvate	-1.48	0.00	-0.56	0.15
acetate	-1.78	0.00	1.32	0.01
malonate	-0.75	0.03	0.78	0.02
2-methylmaleate	-0.76	0.07	0.05	0.92
4-aminobutyrate (GABA)	0.51	0.13	1.19	0.00
N-acetylaspartate	-1.76	0.00	0.70	0.16
**Glycerophospholipid Metabolism**				
glycerone	-1.28	0.01	-0.45	0.53
acetol	-1.04	0.03	0.61	0.43
phosphocholine	1.60	0.15	-2.96	0.05
trimethylamine	-0.87	0.12	1.01	0.03
**Branched-chain Amino Acid Metabolism**				
valine	-1.17	0.00	0.82	0.03
isobutyrate	-1.60	0.07	0.33	0.76
3-hydroxyisovalerate	-0.74	0.15	-0.87	0.03
**Glycine, Serine, and Arginine Metabolism**				
biotin	-1.09	0.06	1.87	0.00
glycolate	-0.78	0.22	1.35	0.02
glycerate-2-phosphate	-0.87	0.03	0.89	0.00
glycerate	0.06	0.93	0.93	0.01
ethylene glycol	-1.34	0.06	-1.39	0.05
glycine	-1.29	0.05	0.33	0.60
sarcosine	0.05	0.93	1.99	0.01
creatine	-0.23	0.84	1.81	0.01
methylguanidine	0.33	0.53	2.24	0.00
guanidoacetate	-0.88	0.12	1.38	0.01
dimethylglycine	0.67	0.31	1.32	0.02
5-aminolevulinate	-0.74	0.04	1.10	0.00
dimethylamine	4.08	0.00	4.78	0.00
**Shikimate Pathway**				
ferulate	-0.81	0.01	1.24	0.00
syringate	0.77	0.42	2.00	0.01
acetylsalicylate	-1.53	0.00	0.42	0.25
3-hydroxyphenylacetate	0.26	0.61	1.33	0.03
5-hydroxyindole-3-acetate	-0.57	0.27	1.01	0.01
xanthurenate	-1.54	0.00	-0.09	0.77
**Pentose Phosphate Pathway**				
pyridoxine	-2.06	0.00	0.38	0.41
caffeine	-1.52	0.03	-0.52	0.37
thymine	-0.47	0.39	1.72	0.00
methylhistidine	-1.37	0.08	0.20	0.75
xanthine	0.48	0.31	1.53	0.00
histamine	-0.85	0.11	1.48	0.00
uridine	-0.92	0.18	1.19	0.05
1,7-dimethylxanthine	-2.25	0.27	1.57	0.01
anserine	-0.81	0.12	0.88	0.05

S1 Table. Metabolites significantly changed upon endosidin-7 treatment in leaves and roots. The difference of metabolite levels in arabidopsis seedlings for endosidin-7 treatment (n=15) versus the control (n=12) is expressed by log2 fold change. The length of the colored bar is proportional to the value, with decreases in blue and increases indicated in orange. A threshold of 1.5 (log2) was used in the multivariate analysis to calculate the *p*-value of significance.
