# Supplementary material for: NMR spectroscopy analysis reveals differential metabolic responses in arabidopsis roots and leaves treated with a cytokinesis inhibitor
Source: PLoS One. 2020 Nov 6;15(11):e0241627. doi: 10.1371/journal.pone.0241627 (PMC7647083; doi:10.1371/journal.pone.0241627)
Supplement: S2 Table — Concentrations (mean±SD) are expressed in mM/g (log2) with reference to the internal standard TSP (n = 12 for controls, n = 15 for endosidin-7 treated). (PDF) [file pone.0241627.s006.pdf]

	Leaves Control	Roots Control	Leaves ES7 Treated	Roots ES7 Treated
**Carbohydrate Metabolism**				
maltose	6.97 ± 0.82	6.76 ± 0.29	2.96 ± 0.82	5.88 ± 0.22
galactitol	7.62 ± 0.33	7.3 ± 0.26	6.53 ± 0.36	7.32 ± 0.18
xylitol	9.24 ± 0.62	7.3 ± 0.23	7.59 ± 0.44	7.87 ± 0.23
glucarate	6.13 ± 0.44	6.08 ± 0.27	5.03 ± 0.29	6.72 ± 0.27
ascorbate	4.7 ± 0.4	3.8 ± 0.33	3.65 ± 0.36	4.61 ± 0.31
fructose	10.69 ± 0.25	10.8 ± 0.17	10.41 ± 0.17	11.71 ± 0.15
fucose	6.25 ± 0.43	5.72 ± 0.18	7.07 ± 0.43	6.66 ± 0.15
sorbitol	9.71 ± 0.23	7.95 ± 0.1	8.66 ± 0.16	10.42 ± 0.23
lactose	6.65 ± 0.34	7.1 ± 0.21	6 ± 0.3	7.85 ± 0.17
sucrose	8.87 ± 0.8	8.12 ± 0.57	8.24 ± 0.62	6.34 ± 0.59
galactarate	5.64 ± 0.3	6.69 ± 0.28	5.27 ± 0.25	7.39 ± 0.24
N-acetylglucosamine	4.18 ± 0.45	4.07 ± 0.26	4.21 ± 0.29	4.68 ± 0.23
**Glycolysis and Krebs Cycle Derivatives**				
pyruvate	7.23 ± 0.29	5.5 ± 0.29	5.75 ± 0.27	4.94 ± 0.25
acetate	5 ± 0.4	3.28 ± 0.36	3.23 ± 0.38	4.6 ± 0.3
malonate	3.83 ± 0.25	4.04 ± 0.24	3.09 ± 0.23	4.82 ± 0.21
2-methylmaleate	3.7 ± 0.31	2.63 ± 0.27	2.94 ± 0.25	2.68 ± 0.43
4-aminobutyrate (GABA)	5.82 ± 0.24	6.98 ± 0.17	6.33 ± 0.19	8.18 ± 0.19
N-acetylaspartate	3.73 ± 0.23	3.14 ± 0.37	1.97 ± 0.29	3.84 ± 0.26
**Glycerophospholipid Metabolism**				
glycerone	2.32 ± 0.35	0.77 ± 0.6	1.03 ± 0.29	0.33 ± 0.42
acetol	4.61 ± 0.36	3.34 ± 0.68	3.57 ± 0.28	3.95 ± 0.36
phosphocholine	2.78 ± 1.02	5.57 ± 0.72	4.37 ± 0.64	2.61 ± 1.44
trimethylamine	1.55 ± 0.37	1.37 ± 0.39	0.68 ± 0.43	2.39 ± 0.26
**Branched-chain Amino Acid Metabolism**				
valine	3.65 ± 0.3	4.67 ± 0.28	2.49 ± 0.25	5.49 ± 0.24
isobutyrate	3.64 ± 0.63	3.09 ± 0.73	2.04 ± 0.63	3.42 ± 0.9
3-hydroxyisovalerate	4.23 ± 0.42	5.18 ± 0.31	3.49 ± 0.31	4.31 ± 0.26
**Glycine, Serine, and Arginine Metabolism**				
biotin	5.87 ± 0.43	3.76 ± 0.27	4.78 ± 0.35	5.64 ± 0.3
glycolate	6.29 ± 0.48	4.92 ± 0.44	5.51 ± 0.42	6.27 ± 0.41
glycerate-2-phosphate	7.75 ± 0.26	6.97 ± 0.17	6.87 ± 0.24	7.86 ± 0.16
glycerate	7.59 ± 0.52	8.51 ± 0.29	7.65 ± 0.37	9.45 ± 0.23
ethylene glycol	10.29 ± 0.53	8.42 ± 0.53	8.95 ± 0.49	7.03 ± 0.46
glycine	9.95 ± 0.49	8.36 ± 0.49	8.66 ± 0.46	8.7 ± 0.44
sarcosine	3.44 ± 0.45	3.85 ± 0.64	3.49 ± 0.41	5.84 ± 0.36
creatine	2.08 ± 0.95	1.49 ± 0.55	1.85 ± 0.73	3.29 ± 0.45
methylguanidine	3.66 ± 0.4	2.12 ± 0.49	3.99 ± 0.36	4.37 ± 0.31
guanidoacetate	9.78 ± 0.43	7.54 ± 0.39	8.9 ± 0.38	8.93 ± 0.34
dimethylglycine	1.51 ± 0.51	1.58 ± 0.45	2.18 ± 0.45	2.9 ± 0.36
5-aminolevulinate	5.3 ± 0.28	4.92 ± 0.25	4.56 ± 0.23	6.03 ± 0.22
dimethylamine	5.52 ± 0.82	2.53 ± 1.07	9.6 ± 0.76	7.31 ± 0.79
**Shikimate Pathway**				
ferulate	2.57 ± 0.24	3.31 ± 0.21	1.76 ± 0.2	4.55 ± 0.27
syringate	0.15 ± 0.74	3.38 ± 0.62	0.62 ± 0.65	5.38 ± 0.49
acetylsalicylate	2.9 ± 0.29	3.22 ± 0.28	1.37 ± 0.28	3.64 ± 0.23
3-hydroxyphenylacetate	2.21 ± 0.35	2.47 ± 0.46	2.47 ± 0.38	3.8 ± 0.38
5-hydroxyindole-3-acetate	2.38 ± 0.38	2.23 ± 0.3	1.81 ± 0.34	3.24 ± 0.21
xanthurenate	2.89 ± 0.31	4.2 ± 0.23	1.35 ± 0.28	4.12 ± 0.19
**Pentose Phosphate Pathway**				
pyridoxine	2.88 ± 0.43	0.16 ± 0.36	0.82 ± 0.36	0.54 ± 0.3
caffeine	3.09 ± 0.57	3.44 ± 0.47	1.58 ± 0.4	2.92 ± 0.36
thymine	2.72 ± 0.42	2.36 ± 0.26	2.25 ± 0.32	4.08 ± 0.2
methylhistidine	4.23 ± 0.63	2.51 ± 0.47	2.86 ± 0.47	2.71 ± 0.43
xanthine	4.22 ± 0.34	3.81 ± 0.35	4.7 ± 0.34	5.34 ± 0.31
histamine	2.77 ± 0.37	2.81 ± 0.33	1.92 ± 0.39	4.29 ± 0.29
uridine	2.81 ± 0.27	3.74 ± 0.38	1.89 ± 0.38	4.93 ± 0.11
1,7-dimethylxanthine	3.32 ± 1.5	1.85 ± 0.43	1.07 ± 1.5	3.42 ± 0.39
anserine	3.01 ± 0.35	3.15 ± 0.34	2.19 ± 0.39	4.04 ± 0.29

S2 Table. Quantification of metabolite levels changes upon endosidin-7 treatment in leaves and roots. Concentrations (mean±SD) are expressed in mM/g (log2) with reference to the internal standard TSP (n = 12 for controls, n = 15 for endosidin-7 treated).
