# Supplementary material for: NMR spectroscopy analysis reveals differential metabolic responses in arabidopsis roots and leaves treated with a cytokinesis inhibitor
Source: PLoS One. 2020 Nov 6;15(11):e0241627. doi: 10.1371/journal.pone.0241627 (PMC7647083; doi:10.1371/journal.pone.0241627)
Supplement: S3 Table — Without endosidin-7 treatment, multivariate analysis does not show significant concentration modulations for these compounds (threshold of 1.5 (log2), p < 0.05). Concentrations (mean±SD) are expressed in mM/g (log2) with reference to the internal standard TSP (n = 3 for each developmental stage). N.D. denotes “not detected”. (PDF) [file pone.0241627.s007.pdf]

	Leaves				Roots			
	4 DAG	5 DAG	6 DAG	10 DAG	4 DAG	5 DAG	6 DAG	10 DAG
**Carbohydrate Metabolism**								
maltose	6.26 ± 0.64	6.05 ± 0.05	6.27 ± 0.43	6.49 ± 0.45	7.42±0.49	2.24±3.88	2.38±4.13	5.99±1.64
galactitol	2.37±4.10	2.62±4.53	7.72±0.37	N.D.	5.09±4.41	7.35±0.23	2.13±3.69	4.90±4.25
xylitol	8.85 ± 0.48	8.75 ± 0.33	7.83 ± 0.16	8.00 ± 0.30	2.48±4.30	4.92±4.31	2.16±3.75	7.48±0.12
glucarate	4.21±3.65	1.95±3.38	N.D.	2.02±3.49	5.78±0.71	5.94±1.43	4.20±3.83	6.36±0.50
ascorbate	3.15±2.73	1.61±2.78	1.51±2.62	N.D.	4.76±0.07	1.92±1.67	0.92±1.60	N.D.
fructose	6.89±5.97	7.13±6.18	3.58±6.21	3.77±6.53	10.70±0.37	10.61±0.17	10.53±0.20	11.34±0.56
fucose	5.99 ± 0.77	6.10 ± 0.12	6.28 ± 0.38	6.70 ± 0.43	5.95±0.66	5.62±0.33	3.61±3.13	5.79±0.20
sorbitol	9.73 ± 0.91	9.46 ± 0.45	8.38 ± 0.23	8.52 ± 0.57	5.44±4.72	2.63±4.56	5.17±4.48	N.D.
lactose	1.98±3.42	4.81±4.21	N.D.	2.08±3.61	7.10±0.13	4.72±4.09	6.52±1.38	7.68±0.30
sucrose	2.79±4.84	6.12±5.34	5.72±4.99	3.12±5.40	11.80±0.49	7.40±0.26	8.20±0.82	5.09±1.51
galactarate	5.88±0.12	4.55±0.99	6.24±0.09	2.12±3.68	6.85±0.82	7.13±0.26	4.00±3.47	6.53±0.14
N-acetylglucosamine	0.89 ± 1.55	N.D.	3.19 ± 2.79	1.49 ± 2.59	4.06±0.65	3.79±1.19	4.01±0.40	4.44±0.79
**Glycolysis and Krebs Cycle Derivatives**								
pyruvate	5.83±1.03	7.49±0.35	7.85±0.16	7.77±0.34	4.91±0.08	5.65±0.94	5.29±0.28	6.14±0.75
acetate	2.67±2.32	3.25±2.95	3.79±3.30	5.30±0.90	1.54±1.43	3.61±0.48	2.51±2.28	4.35±0.20
malonate	3.99±0.34	3.79±0.56	3.45±0.39	2.84±2.70	3.50±0.41	3.87±0.60	4.01±0.18	4.78±0.13
2-methylmaleate	3.81±0.63	2.51±2.33	N.D.	1.09±1.88	3.18±0.90	0.81±1.40	2.37±0.18	0.65±1.13
4-aminobutyrate (GABA)	1.36±2.35	6.22±0.09	N.D.	4.07±3.53	6.78±0.12	6.94±0.04	6.52±0.18	7.69±0.13
N-acetylaspartate	3.76±0.16	1.01±1.74	2.55±2.29	2.63±2.29	N.D.	1.06±1.83	1.00±1.73	1.08±1.88
**Glycerophospholipid Metabolism**								
glycerone	0.50±0.86	1.45±0.69	1.00±1.13	4.01±0.62	0.53 ± 0.50	1.19 ± 0.72	1.64 ± 0.16	2.10 ± 0.68
acetol	5.44±0.41	N.D.	4.04±0.80	1.27±2.20	3.34 ± 0.10	3.46 ± 0.31	3.78 ± 0.38	4.09 ± 0.96
phosphocholine	3.31 ± 1.88	4.28 ± 0.43	4.47 ± 0.59	5.07 ± 0.49	5.92±0.38	N.D.	N.D.	1.50±2.60
trimethylamine	0.19±0.34	0.80±1.08	1.70±0.90	1.44±1.33	-0.33±0.58	1.23±0.27	N.D.	2.32±1.06
**Branched-chain Amino Acid Metabolism**								
valine	3.03±0.50	3.58±3.10	3.09±0.45	2.48±2.15	4.04±0.12	4.66±0.12	4.66±0.22	3.77±3.27
isobutyrate	N.D.	3.07±2.66	1.78±1.56	N.D.	N.D.	0.42±0.73	0.88±1.52	1.80±3.11
3-hydroxyisovalerate	2.98±2.72	4.51±0.66	0.97±1.68	N.D.	5.39±0.17	3.33±2.89	5.00±0.60	5.26±0.46
**Glycine, Serine, and Arginine Metabolism**								
biotin	N.D.	3.88±3.37	1.96±3.39	1.98±3.43	4.39±1.10	2.08±2.00	3.02±0.99	3.06±2.66
glycolate	1.70±2.94	6.56±0.63	6.17±1.24	6.54±0.77	3.44±2.34	4.15±1.61	5.12±0.92	6.96±0.89
glycerate-2-phosphate	7.18 ± 0.58	7.90 ± 0.23	7.63 ± 0.12	7.62 ± 0.12	7.07±0.61	4.54±3.93	6.77±0.31	4.85±4.22
glycerate	2.52±4.37	N.D.	5.07±4.41	N.D.	7.71±0.56	8.52±0.29	5.81±5.04	6.33±5.48
ethylene glycol	12.18±0.05	10.67±0.38	9.23±0.12	9.07±1.41	9.97±0.28	9.14±0.61	8.57±0.63	6.00±1.30
glycine	9.66±2.08	9.67±1.22	10.17±0.13	10.28±0.98	10.07±0.54	8.67±0.57	8.83±0.38	5.88±2.52
sarcosine	3.01±0.40	3.19±0.55	2.41±2.09	2.85±2.49	N.D.	3.73±0.71	0.91±1.58	1.78±3.07
creatine	0.81±1.40	N.D.	0.33±0.58	0.94±1.62	1.18±1.12	1.32±0.56	1.24±0.16	0.71±1.23
methylguanidine	2.99±2.62	2.69±2.82	3.75±0.54	1.54±2.18	1.02±0.11	N.D.	N.D.	3.23±2.85
guanidoacetate	11.10±2.66	10.55±0.42	5.40±4.71	5.56±4.83	7.77±0.55	7.02±0.55	6.97±0.40	8.41±0.61
dimethylglycine	0.38±0.66	0.59±1.75	1.36±1.19	1.69±1.49	0.88±1.52	0.15±1.05	0.66±0.59	3.58±1.91
5-aminolevulinate	3.29±2.90	5.22±0.70	5.62±0.82	3.55±3.14	5.08±0.39	5.03±0.29	4.29±0.90	5.29±1.23
dimethylamine	3.45±0.64	5.51±0.85	5.64±0.17	7.49±0.36	N.D.	1.91±2.10	1.33±2.30	2.65±2.23
**Shikimate Pathway**								
ferulate	0.96±1.66	1.42±1.23	2.10±0.56	3.23±0.58	2.44±0.85	3.49±0.31	3.71±0.25	3.60±0.62
syringate	-0.11±0.19	-0.29±0.62	N.D.	0.05±0.08	1.15±1.28	2.77±1.84	2.46±2.45	4.87±1.92
acetylsalicylate	1.02±1.76	1.65±1.50	2.90±0.46	2.17±2.13	N.D.	2.67±1.21	3.31±0.27	3.67±0.22
3-hydroxyphenylacetate	0.51±0.89	1.20±1.47	1.71±1.49	1.72±1.57	N.D.	1.45±1.63	0.65±1.13	1.18±2.05
5-hydroxyindole-3-acetate	0.75±1.31	1.59±1.38	1.62±1.50	N.D.	0.65±0.69	2.09±0.15	N.D.	3.22±0.46
xanthurenate	0.98±1.69	1.98±1.72	0.79±1.37	2.04±1.86	4.41±0.18	2.50±2.16	4.53±0.27	3.96±0.46
**Pentose Phosphate Pathway**								
pyridoxine	2.05±2.58	3.38±0.62	N.D.	1.28±1.36	-0.02±0.29	-0.21±0.71	0.53±0.58	0.23±0.54
caffeine	0.74±1.29	1.67±2.89	0.23±0.39	3.54±1.22	2.39±1.33	2.94±2.70	3.95±0.62	1.03±1.78
thymine	N.D.	1.78±1.69	0.94±1.63	N.D.	2.24±0.04	1.54±1.33	1.67±1.50	0.85±1.46
methylhistidine	4.02±0.57	N.D.	2.36±3.10	2.08±3.61	1.11±0.45	1.73±1.73	3.86±0.93	2.53±0.68
xanthine	4.06±0.90	4.06±0.37	3.77±1.06	4.98±0.29	3.97±2.48	2.86±0.71	3.34±3.19	3.81±0.84
histamine	1.50±1.46	2.56±0.94	2.17±2.01	2.09±1.83	3.48±1.00	2.17±0.56	2.60±1.29	2.04±2.04
uridine	N.D.	0.79±1.37	N.D.	1.08±1.87	3.07 ± 0.20	3.32 ± 0.39	3.58 ± 0.21	4.14 ± 0.63
1,7-dimethylxanthine	2.05 ± 0.48	1.83 ± 0.14	2.03 ± 0.37	3.19 ± 0.53	1.32±0.38	1.27±1.26	1.65±0.32	3.15±1.55
anserine	3.61±1.44	3.14±0.07	2.68±0.83	1.60±1.39	2.06±0.92	2.61±1.19	3.14±0.52	4.79±0.26

S3 Table. Quantification of metabolite levels changes in leaves and roots during seedling development.

Without endosidin-7 treatment, multivariate analysis does not show significant concentration modulations for these compounds (threshold of 1.5 (log2), $p < 0.05$). Concentrations (mean±SD) are expressed in mM/g (log2) with reference to the internal standard TSP (n = 3 for each developmental stage). N.D. denotes “not detected.”
